# Hesitant Fuzzy Linguistic Preference Utility Set and Its Application in Selection of Fire Rescue Plans

**DOI:** 10.3390/ijerph15040664

**Published:** 2018-04-03

**Authors:** Huchang Liao, Guangsen Si, Zeshui Xu, Hamido Fujita

**Affiliations:** 1 Business School, Sichuan University, Chengdu 610064, China; liaohuchang@scu.edu.cn (H.L.); xuzeshui@263.net (Z.X.); 2 Department of Computer Science and Artificial Intelligence, University of Granada, E-18071 Granada, Spain; liaohuchang@correo.ugr.es; 3 Faculty of Software and Information Science, Iwate Prefectural University, Iwate 020-0193, Japan; hfujita-799@acm.org

**Keywords:** hesitant fuzzy linguistic term set, the prospect theory, linguistic scale function, hesitant fuzzy linguistic preference utility set, hesitant fuzzy linguistic preference utility-TOPSIS method

## Abstract

Hesitant fuzzy linguistic term set provides an effective tool to represent uncertain decision information. However, the semantics corresponding to the linguistic terms in it cannot accurately reflect the decision-makers’ subjective cognition. In general, different decision-makers’ sensitivities towards the semantics are different. Such sensitivities can be represented by the cumulative prospect theory value function. Inspired by this, we propose a linguistic scale function to transform the semantics corresponding to linguistic terms into the linguistic preference values. Furthermore, we propose the hesitant fuzzy linguistic preference utility set, based on which, the decision-makers can flexibly express their distinct semantics and obtain the decision results that are consistent with their cognition. For calculations and comparisons over the hesitant fuzzy linguistic preference utility sets, we introduce some distance measures and comparison laws. Afterwards, to apply the hesitant fuzzy linguistic preference utility sets in emergency management, we develop a method to obtain objective weights of attributes and then propose a hesitant fuzzy linguistic preference utility-TOPSIS method to select the best fire rescue plan. Finally, the validity of the proposed method is verified by some comparisons of the method with other two representative methods including the hesitant fuzzy linguistic-TOPSIS method and the hesitant fuzzy linguistic-VIKOR method.

## 1. Introduction

To address emergency events, a range of decision methods were proposed within different contexts [1,2,3,4]. Although the methods in the above literature made some contributions to address emergency events, few studies considered the decision-makers’ (DMs’) risk preference attitudes in the decision process. In emergency management, the DMs usually have subjective preferences over alternatives and their risk preference attitudes have important impacts on the response of emergency events. In most cases, the DMs cannot utilize crisp numerical values to express complex decision information. Rodríguez et al. [5] proposed the concept of hesitant fuzzy linguistic term set (HFLTS), which provides a flexible tool for the DMs to elicit uncertain information. With the mathematical definition of HFLTS given by Liao et al. [6], a lot of multiple attribute decision making (MADM) methods have been proposed under hesitant fuzzy linguistic environment [7,8,9]. However, the operations of linguistic terms in these methods are conducted based on the subscripts of the linguistic terms. In this sense, the decision results obtained by operating over the subscripts may be inconsistent with the DMs’ cognition given that the semantics of the original linguistic terms are lost.

Different linguistic scale functions were introduced [10,11]. Silva and Morais [12] constructed a linguistic scale function with five levels to evaluate the priority of infrastructure works, where the function associated with each term was obtained by linear regression. Considering that the normal linguistic scale cannot model subjective judgments, Peng and Zheng [13] introduced different unbalanced linguistic scale sets. In addition, Dong et al. [14] proposed a linguistic scale function to transform the linguistic term into real numbers. However, the linguistic scale functions listed in the above literature cannot accurately reflect the DMs’ subjective feelings and risk preference attitudes.

The expected utility theory assumes that people are completely rational. However, in practical decision process, people tend to make decision according to their risk preference attitudes as well as the way of thinking. Kahneman and Tversky [15] first developed the prospect theory to overcome the limitations of the expected utility theory. Subsequently, the cumulative prospect theory [16] was proposed to assign different weighting functions to the gains and losses, respectively. Compared with the expected utility theory, the cumulative prospect theory value function can accurately reflect the DMs’ sensitivity towards the gains and losses. As Liu et al. [17] noted, a decision based on the prospect theory is more in line with people’s decision behavior than the expected utility theory. Many scholars proposed different MADM methods [18,19] where the prospect theory is used to reflect the DMs’ subjective feelings. Additionally, Wang et al. [20] pointed that the DMs’ psychological behavior has great effects on the decision result and developed a MADM method to solve the case concerning the barrier lake emergency. Using the prospect theory to consider the DMs’ decision behavior, Qin et al. [21] extended the VIKOR method within the interval type-2 fuzzy context. To accurately reflect the DMs’ risk preference attitudes, by integrating the trapezoidal fuzzy numbers and the value function of prospect theory, Krohling and de Souza [22] developed a novel fuzzy TODIM method.

Motivated by the above achievements and based on the cumulative prospect theory value function, in this paper, we propose a new linguistic scale function to transform the semantics into the corresponding linguistic preference values. In this sense, we can flexibly express the semantics corresponding to the linguistic terms in the HFLTS and facilitate the operation process. Moreover, to accurately reflect the DMs’ subjective feelings, we introduce a new information representation tool named the hesitant fuzzy linguistic preference utility set (HFLPUS) where different parameters can be utilized to reflect the DMs’ risk preference attitudes. Specifically, this paper intends to achieve the following novel contributions:

(1) Based on the cumulative prospect theory value function, we propose a novel linguistic scale function to transform the semantics into the linguistic preference values, which can accurately reflect the DMs’ subjective feelings. Besides, different parameters can be used to reflect the DMs’ risk preference attitudes. With this model, the decision results that are consistent with the DMs’ cognition can be obtained.
(2) To flexibly express semantics, based on the proposed linguistic scale function, we propose the HFLPUS to represent the DMs’ cognition. In addition, to facilitate the calculations and comparisons, we introduce the distance measures and comparison law for HFLPUSs, where the operations are conducted according to the DMs’ subjective feelings rather than directly based on the subscripts of linguistic terms.
(3) To overcome the instability of the subjective weight-determining method, under the linguistic preference value circumstance, we propose a method to obtain the objective weights based on the diversity of attribute information. To solve the MADM problem with HFLPUSs, we then develop a hesitant fuzzy linguistic preference utility-TOPSIS (HFLPU-TOPSIS) method and apply it to address a case concerning the selection of fire rescue plans.

The rest of this paper is organized as follows: Section 2 reviews some relevant knowledge regarding to the fuzzy linguistic approach, the HFLTS and the cumulative prospect theory value function. In Section 3, based on the cumulative prospect theory value function and HFLTSs, we present a linguistic scale function and the concept of the HFLPUS. Some distance measures and comparison laws for HFLPUSs are given in this section as well. Section 4 proposes an objective weight-determining method based on the diversity of attribute information. After that, the HFLPU-TOPSIS method is proposed to cope with emergency events. Section 5 applies the proposed HFLPU-TOPSIS method to address a case concerning the selection of fire rescue plans. Then, some comparisons with other two representative MADM methods are conducted to verify the validity of the proposed method. The paper ends in Section 6.

## 2. Preliminaries

To have a better understanding of the linguistic scale function and the HFLPUS, this section reviews some relevant knowledge, including the fuzzy linguistic approach, the HFLTS and the prospect theory.

### 2.1. Fuzzy Linguistic Approach

In general, due to the complexity of the problems, it is difficult for the DMs to give precise numerical values for fuzzy information. Zadeh [23] proposed the fuzzy linguistic approach, which considers the linguistic information as the values of linguistic variables. The linguistic variables are composed of linguistic descriptors and the corresponding semantics [24]. Afterwards, different methods were proposed to select the linguistic description operators and give their corresponding semantics [5,24,25]. Let $S=\{s_{i}|i=0,1,2,\ldots ,g\}$ be a finite and totally ordered discrete linguistic term set with odd cardinality, where $s_{i}$ represents a possible value for a linguistic variable, g+1 is the granularity of the linguistic term set and S satisfies the following conditions: (1) The set is ordered: $s_{i}\geq s_{j}$ if i≥j; (2) Negation operator: $neg(s_{i})=s_{j}$, i+j=g; (3) Max operator: $max(s_{i},s_{j})=s_{i}$ if $s_{i}\geq s_{j}$.

Xu [26] proposed a subscript-symmetric linguistic term set as:

(1) $$S=\left\{s_{t}|t=-\tau ,\ldots ,0,\ldots ,\tau \right\}$$

where $s_{0}$ represents a possible value of the semantic “indifference,” especially, $s_{-\tau }$ and $s_{\tau }$ are the lower and upper bounds of S, respectively. τ is a possible integer. In addition, to preserve all the given information, Xu [26] further extended the linguistic term set S, shown as Equation (1), to a continuous linguistic term set as:

(2) $$\bar{S}=\left\{s_{\varsigma }|\varsigma \in \left[-q,q\right]\right\}$$

where q(q>τ) is a sufficiently large positive integer. If $s_{\varsigma }\in S$, then $s_{\varsigma }$ is called an original linguistic term; otherwise, $s_{\varsigma }$ is called a virtual linguistic term. For any two linguistic terms $s_{a},s_{b}\in \bar{S}$ and $\rho ,\rho_{1},\rho_{2}\in [0,1]$, the following operation laws were introduced [27]: (1) $s_{a}⊕s_{b}=s_{a+b}$; (2) $\rho s_{a}=s_{\rho a}$; (3) $\rho \left(s_{a}⊕s_{b}\right)=\rho s_{a}⊕\rho s_{b}$.

### 2.2. Hesitant Fuzzy Linguistic Term Set

Let $S=\{s_{0},\ldots ,s_{g}\}$ be a linguistic term set with odd cardinality. Rodríguez et al. [5] proposed the concept of the HFLTS as an ordered finite subset of the consecutive linguistic terms of S. The HFLTS allows the DMs to use several possible linguistic terms to elicit uncertain decision information. Later, Liao et al. [6] redefined the HFLTS mathematically below:

**Definition 1** **[6].** *Let x∈X be fixed and $S=\{s_{t}|t=-\tau ,\ldots ,-1,0,1,\ldots ,\tau \}$ be a linguistic term set. A HFLTS, $H_{S}$, is in mathematical term of*

(3) $$H_{S}=\{<x,h_{S}\left(x\right)>|x\in X\}$$

*where $h_{S}\left(x\right)$ denotes the possible degrees of the linguistic variable x to the linguistic term set S. For convenience, $h_{S}\left(x\right)$ is called hesitant fuzzy linguistic element (HFLE).*

### 2.3. Cumulative Prospect Theory Value Function

Kahneman and Tversky [15] proposed the prospect theory, which can reflect the DMs’ sensitivity towards the gains and losses. Afterwards, the cumulative prospect theory was proposed [16], which allows the gains and losses to have different weighting functions expressed in the form of a piecewise function as:

(4) $$V\left(x\right)=\left\{ \begin{array}{ll} x^{\alpha } & x\geq 0\\ -\lambda {\left(-x\right)}^{\beta } & x<0 \end{array}\right.$$

where α(0≤α<1) and β(0≤β<1) are risk attitude coefficients related to the gains and losses, respectively. x=0 is the decision reference point, that is, the DMs’ psychological balance reference point. λ is the risk aversion parameter, which means that the losses function is steeper than the gains function. Especially, λ>1 represents the losses aversion.

**Figure 1:**
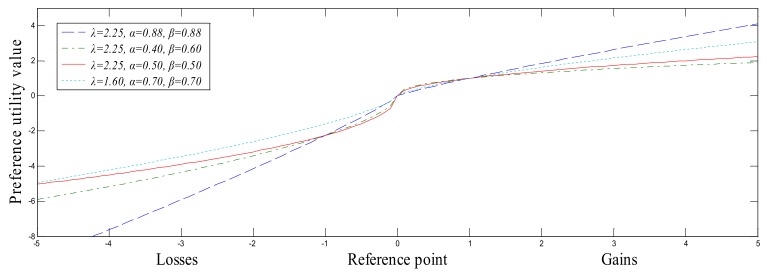
The cumulative prospect theory value function.

By Equation (4), we can see that the cumulative prospect theory value function is divided into the gains domain and losses domain, where the DMs are characterized by the risk aversion and the risk preference, respectively. Especially, when α=β=0.88, λ=2.25, the experimental data is consistent with the empirical data [16]. In addition, the DMs’ negative utility on the losses is greater than the positive utility of gains, shown as Figure 1.

## 3. Linguistic Scale Function and HFLPUS

In this section, inspired by the cumulative prospect theory value function, we propose a linguistic scale function to transform the semantics corresponding to linguistic terms in the HFLTS into the linguistic preference values. According to that, we propose the HFLPUS to flexibly express the semantics and obtain the decision results that are consistent with the DMs’ cognition.

### 3.1. Linguistic Scale Function

The linguistic scale function provides a scientific basis for the DMs to make decision by combining the qualitative linguistic evaluation information and the quantitative information. Moreover, due to the complexity and uncertainty of practical problems, there is no linguistic evaluation scale that is suitable for all problems. To make judgment according to the DMs’ risk preference attitudes, it is necessary to introduce a novel linguistic scale function.

As presented in Figure 1, the same subjective feelings may correspond to different preference utility values which implies that the DMs’ risk preference attitudes are different. In general, due to the DMs’ different knowledge and experience, for the same object, different DMs may give different evaluation information. For example, when evaluating the performance of a car, some experts may deem that it is “very good” while some may think it is “good”. In addition, the DMs may use different scores to express the same subjective feeling “very good”, such as 90 or 80. In this sense, the semantics corresponding to different linguistic terms cannot reflect the DMs’ subjective feelings. Thus, it is necessary to transform the semantics corresponding to linguistic terms into the linguistic preference values.

Inspired by the cumulative prospect theory value function shown as Figure 1, we propose a new linguistic scale function to assign different linguistic preference values to the semantics corresponding to linguistic terms, which can accurately reflect the DMs’ subjective feelings.

**Definition** **2.** *Let $S=\left\{s_{i}|i=0,1,2,\ldots 2t\right\}$ be a linguistic term set with odd cardinality, $\theta_{i}\in R$(i=0,1,2…,2t) be the linguistic preference values corresponding to the semantics of linguistic terms. Then, a linguistic scale function U can be expressed as:*

(5) $$U\left(s_{i}\right)=\theta_{i}=\left\{ \begin{array}{ll} t-\lambda {\left(t-i\right)}^{\beta } & i=0,1,2,\ldots ,t\\ t+{\left(i-t\right)}^{\alpha } & i=t+1,t+2,\ldots ,2t \end{array}\right.$$

*where α(0≤α<1) and β(0≤β<1) are the DMs’ risk preference attitude coefficients corresponding to the gains and losses, respectively. λ(λ>1) is the risk aversion parameter and t is a sufficiently large positive integer. In particular, the linguistic preference value $\theta_{t}$ denotes the semantic of “indifference,” and the remainder of them are placed symmetrically around it. $\theta_{0}$ and $\theta_{2t}$ are the lower and upper bounds of the linguistic preference values.*

**Figure 2:**
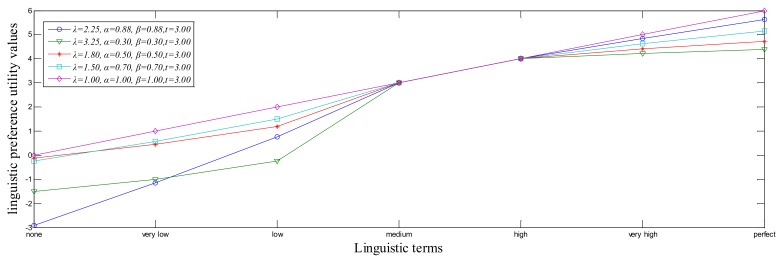
A set of seven linguistic terms with their linguistic preference utility values.

In addition, by Equation (5), different risk preference parameters can be used to transform the semantics into their corresponding linguistic preference values and also reflect different DMs’ risk preference attitudes. For example, for a set of seven linguistic term set $S=\{s_{0}=none,s_{1}=very\;low,s_{2}=low,s_{3}=medium,s_{4}=high,s_{5}=very\;high,s_{6}=perfect\}$, by Equation (5), the obtained linguistic preference values are shown as Figure 2.

It is noted that U is a strictly monotonically increasing and continuous function, where the absolute deviation of linguistic preference values between two adjacent subjective feelings gradually decreases. Let $\bar{S}=\left\{s_{i}|i\in [0,2t]\right\}$ be a continuous linguistic term set. To preserve all the given information, the linguistic scale function U can be extended to a strictly monotonically increasing and continuous linguistic scale function $U^{*}:\bar{S}\to R$, which satisfies $U^{*}\left(s_{i}\right)=\theta_{i}$ and $U^{*-1}$ is the reverse function of $U^{*}$. Then, the linguistic terms $s_{i}\left(0\leq i\leq 2t\right)$ corresponding to the linguistic preference values $\theta_{i}\left(i\in [0,2t]\right)$ can be calculated as:

(6) $$U^{*-1}\left(\theta_{i}\right)=\left\{ \begin{array}{ll} s_{t-{\left(\frac{t-\theta_{i}}{\lambda }\right)}^{\frac{1}{\beta }}} & \theta_{i}\in [t-\lambda t^{\beta },t]\\ s_{t+{\left(\theta_{i}-t\right)}^{\frac{1}{\alpha }}} & \theta_{i}\in (t,t+t^{\alpha }] \end{array}\right.$$

What is more, according to the operations of linguistic terms [27], the aggregated results of linguistic terms are usually not interpretable, For example, given that $S=\{s_{0}=none,s_{1}=very\;low,s_{2}=low,s_{3}=medium,s_{4}=high,s_{5}=very\;high,s_{6}=perfect\}$ $s_{2}=low,s_{3}=medium,$ we have $s_{2}⊕s_{4}$ $=s_{6}$, which means that the aggregated result of linguistic terms “*low*” and “*high*” is “*perfect*.” Nevertheless, this does not conform to the DMs’ cognition. For potential applications, based on the proposed linguistic scale function, we then define some novel operations to obtain the aggregated results that conform to the DMs’ cognition.

**Definition** **3.** *Let $\bar{S}=\left\{s_{i}|i\in [0,2t]\right\}$ be a continuous linguistic term set and $U\left(s_{i}\right)\left(i=a,b\right)$ be the linguistic preference values of the semantics corresponding to the linguistic terms $s_{i}\left(i=a,b\right)$ in $\bar{S}$. Then,*

(1) $neg(s_{a})=U^{*-1}\left(U\left(s_{2t}\right)-U\left(s_{a}\right)\right)$;
(2) $s_{a}⊕s_{b}=U^{*-1}\left(U\left(s_{a}\right)+U\left(s_{b}\right)\right)$;
(3) $\vartheta s_{a}=U^{*-1}\left(\vartheta U\left(s_{a}\right)\right)$, where ϑ∈[0,1].

**Example** **1.** Let $\bar{S}=\left\{s_{i}|0\leq i\leq 6\right\}$ be a continuous linguistic term set and $s_{2},s_{4}\in \bar{S}$ be two linguistic terms in $\bar{S}$. Suppose that ρ=0.8, α=β=0.88, λ=2.25. By Definition 3, we have $neg(s_{2})=s_{5.05}$, $s_{2}⊕s_{4}=s_{4.89}$, $0.8s_{2}=s_{1.92}$.

### 3.2. Hesitant Fuzzy Linguistic Term Preference Utility Set

As it is stated in the introduction, the HFLTSs provide an effective tool for the DMs to elicit uncertain decision information. However, according to the above analysis, the semantics corresponding to linguistic terms in the HFLTS cannot accurately reflect the DMs’ subjective feelings. Thus, the decision results obtained by using the HFLTSs do not conform to the DMs’ cognition. To address this issue, according to the proposed linguistic scale function shown as Equation (5), we propose the HFLPUS to flexibly represent the semantics of linguistic terms in the HFLTS.

**Definition** **4.** *Let $S=\left\{s_{i}|i=0,1,2,\ldots 2t\right\}$ be the linguistic term set and $H_{S}=\{<x,h_{S}\left(x\right)>|x\in X\}$ with $h_{S}=\cup_{s_{i}\in h_{S}}\left\{s_{i}|i=1,2,\ldots ,\#h_{S}\right\}$ be the HFLTS on S. U is the linguistic scale function defined in Definition 2. Then, the HFLPUS ${\bar{U}}_{h_{S}}$ can be represented in the mathematical form of*

(7) $${\bar{U}}_{h_{S}}=\cup_{s_{i}\in h_{S}}\left\{U\left(s_{i}\right)|i=1,2,\ldots \#h_{S}\right\}$$

*where $\#h_{S}$ is the number of linguistic terms in $h_{S}$ and $U(s_{i})(i=1,2,\ldots \#h_{S})$ are the linguistic preference values, denoting the semantic values of the linguistic terms in $h_{S}$.*

Regarding to the linguistic term set $S=\{s_{0}=none,s_{1}=very\;low,$ $s_{2}=low,s_{3}=medium,$ $s_{4}=high,$ $s_{5}=very\;high,$ $s_{6}=perfect\}$, we can flexibly express the assessment of a PhD candidate’s academic potential as “between medium and very high” with the HFLE $h_{S}=\{s_{3},s_{4},s_{5}\}$. By the proposed linguistic scale function, $h_{S}$ can be transformed to the HFLPUS ${\bar{U}}_{h_{S}}=\{U(s_{3}),U(s_{4}),U(s_{5})\}$. Then, according to the expert’s risk preference attitudes, the linguistic preference values $U(s_{3})$, $U(s_{4})$, $U(s_{5})$ can be used to denote the possible membership degrees for the assessment of a PhD candidate’s academic potential.

By the above analysis, we know that either the HFLTS [5] or the hesitant fuzzy set (HFS) [28] can be used to represent the qualitative and quantitative information. However, the proposed HFLPUS integrates both the qualitative and quantitative information by the linguistic scale function, which can not only increase the richness of decision information representation but also obtain the decision results that conform to the DMs’ cognition. Meanwhile, with the HFLPUS, different parameters can be used to reflect the DMs’ risk preference attitudes. Moreover, the DMs can simplify the calculation process and flexibly express the semantics corresponding to linguistic terms in the HFLTS.

For potential applications, it is necessary to introduce some operations for HFLPUSs as follows:

**Definition** **5.** *Let ${\bar{U}}_{h_{S}}$, ${\bar{U}}_{h_{S}^{1}}$ and ${\bar{U}}_{h_{S}^{2}}$ be three HFLPUSs. Then the following operations hold:*

(1) Lower bound: ${\bar{U}}_{h_{S}^{-}}=min\left(U\left(s_{i}\right)\right)=U\left(s_{j}\right)$, $s_{i}\in h_{S}$ and i≥j, ∀i;
(2) Upper bound: ${\bar{U}}_{h_{S}^{+}}=max\left(U\left(s_{i}\right)\right)=U\left(s_{j}\right)$, $s_{i}\in h_{S}$ and i≤j, ∀i;
(3) ${\bar{U}}_{h_{S}^{1}}\cup {\bar{U}}_{h_{S}^{2}}=\left\{U\left(s_{t}\right)|U\left(s_{t}\right)\in {\bar{U}}_{h_{S}^{1}}\;or\;{\bar{U}}_{h_{S}^{2}}\right\}$;
(4) ${\bar{U}}_{h_{S}^{1}}\cap {\bar{U}}_{h_{S}^{2}}=\left\{U\left(s_{t}\right)|U\left(s_{t}\right)\in {\bar{U}}_{h_{S}^{1}}\;and\;{\bar{U}}_{h_{S}^{2}}\right\}$.

Inspired by the score and variance functions of HFLTSs [8], we define the score and variance functions for HFLPUSs to compare two HFLPUSs:

**Definition** **6.** For a HFLPUS ${\bar{U}}_{h_{S}}=\cup_{s_{i}\in h_{S}}\left\{U\left(s_{i}\right)|i=1,2,\ldots \#h_{S}\right\}$ where $\#h_{S}$ is the number of linguistic terms in the HFLE $h_{S}$, $\rho \left({\bar{U}}_{h_{S}}\right)=\frac{1}{\#h_{S}}\sum_{s_{i}\in h_{S}}U\left(s_{i}\right)$ is called the score of ${\bar{U}}_{h_{S}}$ and $\sigma \left({\bar{U}}_{h_{S}}\right)=\frac{1}{\#h_{S}}\sqrt{\sum_{s_{j},s_{k}\in h_{S}}{\left(U\left(s_{j}\right)-U\left(s_{k}\right)\right)}^{2}}$ is called the variance of ${\bar{U}}_{h_{S}}$.

For two HFLPUSs ${\bar{U}}_{h_{S}^{1}}$ and ${\bar{U}}_{h_{S}^{2}}$, if $\rho ({\bar{U}}_{h_{S}^{1}})>\rho ({\bar{U}}_{h_{S}^{2}})$, then ${\bar{U}}_{h_{S}^{1}}>{\bar{U}}_{h_{S}^{2}}$; else if $\rho ({\bar{U}}_{h_{S}^{1}})=\rho ({\bar{U}}_{h_{S}^{2}})$, then (1) if $\sigma ({\bar{U}}_{h_{S}^{1}})<\sigma ({\bar{U}}_{h_{S}^{2}})$, ${\bar{U}}_{h_{S}^{1}}>{\bar{U}}_{h_{S}^{2}}$; (2) if $\sigma ({\bar{U}}_{h_{S}^{1}})=\sigma ({\bar{U}}_{h_{S}^{2}})$, ${\bar{U}}_{h_{S}^{1}}={\bar{U}}_{h_{S}^{2}}$.

For two HFLTSs with different numbers of linguistic terms, we can add the linguistic terms into the shorter one until both of them have the same length [6]. However, the operations are conducted over the subscripts of linguistic terms, which may cause the information changed between the obtained HFLTS and the original one. To add the element reasonably according to the DMs’ subjective feelings, we extend the shorter HFLPUS with the linguistic preference values.

**Definition** **7.** *Let ${\bar{U}}_{h_{S}}=\cup_{s_{i}\in h_{S}}\left\{U\left(s_{i}\right)|i=1,2,\ldots \#h_{S}\right\}$ be a HFLPUS with $\#h_{S}$ being the number of linguistic terms in the HFLE $h_{S}$, ${\bar{U}}_{h_{S}^{+}}$ and ${\bar{U}}_{h_{S}^{-}}$ be the maximum and minimum linguistic preference values in ${\bar{U}}_{h_{S}}$, respectively and ς(0≤ς≤1) be an optimized parameter. Then, we extend the shorter HFLPUS by adding the linguistic preference value as:*

(8) $$s_{\psi }=\varsigma {\bar{U}}_{h_{S}^{+}}+\left(1-\varsigma \right){\bar{U}}_{h_{S}^{-}}$$

When ς=1 and ς=0, the added linguistic preference values correspond with the optimism and pessimism rules, respectively. Without loss of any generality, we assume that the DMs are risk neutral. So we take ς=1/2 in the following operations:

**Example** **2.** Let $h_{S}^{1}=\{s_{0},s_{1},s_{2},s_{3}\}$ and $h_{S}^{2}=\{s_{0},s_{1},s_{2},s_{3},s_{4}\}$ be two HFLEs on S. Suppose that ς=1/2, t=3, α=β=0.88, λ=2.25. By Equation (5), we can obtain their corresponding HFLPUSs $U_{h_{S}^{1}}=\{-2.92,-1.14,0.75,3.00\}$ and $U_{h_{S}^{2}}=\{-2.92,-1.14,0.75,3.00,4.00\}$, respectively. For the shorter HFLPUS $U_{h_{S}^{1}}$, by Equation (8), the linguistic preference value 0.04 will be added to it. Meanwhile, by the extension method of HFLTSs [6], the linguistic term $s_{1.5}$ will be added to $h_{S}^{1}$. However, the operation generating the linguistic term $s_{1.5}$ is based on the numerical operation of subscripts of the linguistic terms $s_{0}$ and $s_{3}$, which does not take into account the DMs’ risk preference attitudes.

The distance measure is widely used to measure the deviation between different elements. Liao et al. [7] defined a range of distance measures between HFLTSs. However, these operations are conducted by the subscripts of linguistic terms in HFLTSs. In this sense, the obtained results may be inconsistent with the DMs’ cognition. To address this issue, we define the Euclidean distance measure between HFLPUSs. To do so, we first add the linguistic preference value generated by Equation (8) to the shorter HFLPUS and then compute the distance between HFLPUSs.

**Definition** **8.** *Let ${\bar{U}}_{h_{S}^{1}}=\cup_{s_{i}^{1}\in h_{S}^{1}}\left\{U\left(s_{i}^{1}\right)|i=1,2,\ldots \#h_{S}^{1}\right\}$ and ${\bar{U}}_{h_{S}^{2}}=\cup_{s_{i}^{2}\in h_{S}^{2}}\left\{U\left(s_{i}^{2}\right)|i=1,2,\ldots \#h_{S}^{1}\right\}$ be two HFLPUSs with $\#{\bar{U}}_{h_{S}^{1}}=\#{\bar{U}}_{h_{S}^{2}}=I$. Suppose that the linguistic preference values $U\left(s_{i}^{v}\right)$ in ${\bar{U}}_{h_{S}^{v}}\left(v=1,2\right)$ are placed in ascending order. Then the Euclidean distance between them can be defined as:*

(9) $$d_{ed}\left({\bar{U}}_{h_{S}^{1}},{\bar{U}}_{h_{S}^{2}}\right)={\left(\frac{1}{I}\sum_{i=1}^{I}{\left(\frac{\left|U\left(s_{i}^{1}\right)-U\left(s_{i}^{2}\right)\right|}{U\left(s_{2t}\right)-U\left(s_{0}\right)}\right)}^{2}\right)}^{1/2}$$

**Theorem** **1.** *The Euclidean distance measure $d_{ed}\left({\bar{U}}_{h_{S}^{1}},{\bar{U}}_{h_{S}^{2}}\right)$ satisfies the following properties:*

(1) $0\leq d_{ed}\left({\bar{U}}_{h_{S}^{1}},{\bar{U}}_{h_{S}^{2}}\right)\leq 1$;
(2) $d_{ed}\left({\bar{U}}_{h_{S}^{1}},{\bar{U}}_{h_{S}^{2}}\right)=0$ *iff* ${\bar{U}}_{h_{S}^{1}}={\bar{U}}_{h_{S}^{2}}$;
(3) $d_{ed}\left({\bar{U}}_{h_{S}^{1}},{\bar{U}}_{h_{S}^{2}}\right)=d_{ed}\left({\bar{U}}_{h_{S}^{2}},{\bar{U}}_{h_{S}^{1}}\right)$.

**Example** **3.** Let $h_{S}^{1}=\{s_{0},s_{1},s_{2}\}$ and $h_{S}^{2}=\{s_{4},s_{5},s_{6}\}$ be two HFLEs on S. Suppose that ς=1/2, t=3, α=β=0.88, λ=2.25. By Equation (5), we can obtain their corresponding HFLPUSs $U_{h_{S}^{1}}=\{-2.92,-1.14,0.75\}$ and $U_{h_{S}^{2}}=\{4,4.84,5.63\}$, respectively. By Equation (9), the Euclidean distance between ${\bar{U}}_{h_{S}^{1}}$ and ${\bar{U}}_{h_{S}^{2}}$ is obtained as 0.70. However, by the distance measure between HFLTSs [7], the Euclidean distance between $h_{S}^{1}$ and $h_{S}^{2}$ is obtained as 0.67. Similar to the extension method of HFLTSs [6], the Euclidean distance of HFLTSs also does not take into account the DMs’ risk preference attitudes.

## 4. A MADM Method with HFLPUSs

This section develops a HFLPU-TOPSIS method to tackle the emergency management problem. To overcome the instability of subjective weights, we develop a method to determine objective weights of attributes according to the diversity of attribute information.

### 4.1. A Method to Determine Objective Weights

As we know, information diversity is one of the core elements in tackling emergency events. The dispersion degree is an important index for measuring the amount of information that the attribute provides. In general, the greater the dispersion degree of the attribute over the remaining attributes is, the more amount of information the attribute provides. Thus, this attribute should be assigned a larger weight.

For the judgments expressed in HFLPUSs, we can calculate the diversity degree $\psi_{lk}$ between the attributes $C_{l}$ and $C_{k}$ and obtain

(10) $$\psi_{lk}=\frac{1}{m}\sum_{i=1}^{m}d\left({\bar{U}}_{h_{S}^{il}},{\bar{U}}_{h_{S}^{ik}}\right),l,k=1,2,\ldots ,n$$

Let $\psi_{l}=\sum_{k=1}^{n}\psi_{lk}$ be the deviation degree of the attribute $C_{l}$ over the remaining attributes. By the above analysis, the larger $\psi_{l}$ is, the farther the distance between the attribute $C_{l}$ and the remaining attributes is. Thus, we should assign a large weight to $C_{l}$, which can be calculated as:

(11) $$\omega_{l}=\frac{\psi_{l}}{\sum_{i=1}^{n}\psi_{i}},l=1,2,\ldots ,n$$

### 4.2. The HFLPU-TOPSIS Method

The HFLTS is an effective tool to elicit uncertain decision information and different methods [5,6,7,8,9,10] have been developed to address MADM problems. However, the semantics corresponding to linguistic terms in HFLTSs cannot accurately reflect the DMs’ subjective feelings. Thus, the decision results obtained with the HFLTSs may be inconsistent with the DMs’ cognition. To overcome this limitation, we develop a HFLPU-TOPSIS method to obtain the decision result that conforms to the DMs’ cognition.

A MADM problem within the HFLPUSs is described below: Let $A=\left\{A_{1},A_{2},\ldots ,A_{m}\right\}$ be a discrete collection of alternatives, $C=\left\{C_{1},C_{2},\ldots ,C_{n}\right\}$ be a discrete collection of attributes whose weight vector is $\omega ={\left(\omega_{1},\omega_{2},\ldots ,\omega_{n}\right)}^{T}$ with $\omega_{j}\geq 0$, j=1,2,…,n, $\sum_{j=1}^{n}\omega_{j}=1$ and the linguistic term set $S=\left\{s_{i}|i=0,1,2,\ldots ,2t\right\}$ is established for evaluation. In this paper, we apply the context-free grammar [5] to elicit emergency decision information. For application, the general procedure of the HFLPU-TOPSIS method involves the following steps:

Step 1. Define the decision alternatives $A=\left\{A_{1},A_{2},\ldots ,A_{m}\right\}$, the attributes $C=\left\{C_{1},C_{2},\ldots ,C_{n}\right\}$ and the weights $\omega ={\left(\omega_{1},\omega_{2},\ldots ,\omega_{n}\right)}^{T}$ with respect to the attributes $C=\left\{C_{1},C_{2},\ldots ,C_{n}\right\}$ for a MADM problem.

Step 2. Define the semantics and syntax of linguistic term set for attributes, based on which, the DMs give the linguistic evaluation values of the alternatives with respect to the attributes, which are represented as ${\left(ll_{ij}\right)}_{m\times n}$ and then a linguistic judgment matrix Q can be established as:

(12) $$Q={\left(ll_{ij}\right)}_{m\times n}=\left[\begin{array}{cccc} ll_{11} & ll_{12} & \cdots & ll_{1n}\\ ll_{21} & ll_{22} & \cdots & ll_{2n}\\ \vdots & \vdots & \ddots & \vdots \\ ll_{m1} & ll_{m2} & \cdots & ll_{mn} \end{array}\right]$$

Step 3. Transform the linguistic expressions ${\left(ll_{ij}\right)}_{m\times n}$ into the corresponding HFLEs ${\left(h_{S}^{ij}\right)}_{m\times n}$ via the transformation function [5]. Then, the hesitant fuzzy linguistic judgment matrix H can be established as:

(13) $$H={\left(h_{S}^{ij}\right)}_{m\times n}=\left[\begin{array}{cccc} h_{S}^{11} & h_{S}^{12} & \cdots & h_{S}^{1n}\\ h_{S}^{21} & h_{S}^{22} & \cdots & h_{S}^{2n}\\ \vdots & \vdots & \ddots & \vdots \\ h_{S}^{m1} & h_{S}^{m2} & \cdots & h_{S}^{mn} \end{array}\right]$$

Step 4. Transform the hesitant fuzzy linguistic judgment matrix H into the corresponding HFLPU judgment matrix $\bar{U}$ by the linguistic scale function shown as Equation (5).

(14) $$\bar{U}={\left({\bar{U}}_{h_{S}^{ij}}\right)}_{m\times n}=\left[\begin{array}{cccc} {\bar{U}}_{h_{S}^{11}} & {\bar{U}}_{h_{S}^{12}} & \cdots & {\bar{U}}_{h_{S}^{1n}}\\ {\bar{U}}_{h_{S}^{21}} & {\bar{U}}_{h_{S}^{22}} & \cdots & {\bar{U}}_{h_{S}^{2n}}\\ \vdots & \vdots & \ddots & \vdots \\ {\bar{U}}_{h_{S}^{m1}} & {\bar{U}}_{h_{S}^{m2}} & \cdots & {\bar{U}}_{h_{S}^{mn}} \end{array}\right]$$

Step 5. Calculate the Euclidean distance between each evaluation value in $\bar{U}$ and the HFLPU positive ideal solution (HFLPU-PIS) $A^{+}$ and the HFLPU negative ideal solution (HFLPU-NIS) $A^{-}$ by Equation (9). Then, the positive ideal separation matrix $D^{+}$ and the negative ideal separation matrix $D^{-}$ can be established as:

(15) $$D^{+}=\left[\begin{array}{cccc} d\left({\bar{U}}_{h_{S}^{11}},{\bar{U}}_{h_{S}^{1}}^{+}\right) & d\left({\bar{U}}_{h_{S}^{12}},{\bar{U}}_{h_{S}^{2}}^{+}\right) & \cdots & d\left({\bar{U}}_{h_{S}^{1n}},{\bar{U}}_{h_{S}^{n}}^{+}\right)\\ d\left({\bar{U}}_{h_{S}^{21}},{\bar{U}}_{h_{S}^{1}}^{+}\right) & d\left({\bar{U}}_{h_{S}^{22}},{\bar{U}}_{h_{S}^{2}}^{+}\right) & \cdots & d\left({\bar{U}}_{h_{S}^{2n}},{\bar{U}}_{h_{S}^{n}}^{+}\right)\\ \vdots & \vdots & \ddots & \vdots \\ d\left({\bar{U}}_{h_{S}^{m1}},{\bar{U}}_{h_{S}^{1}}^{+}\right) & d\left({\bar{U}}_{h_{S}^{m2}},{\bar{U}}_{h_{S}^{m}}^{+}\right) & \cdots & d\left({\bar{U}}_{h_{S}^{mn}},{\bar{U}}_{h_{S}^{n}}^{+}\right) \end{array}\right]$$

(16) $$D^{-}=\left[\begin{array}{cccc} d\left({\bar{U}}_{h_{S}^{11}},{\bar{U}}_{h_{S}^{1}}^{-}\right) & d\left({\bar{U}}_{h_{S}^{12}},{\bar{U}}_{h_{S}^{2}}^{-}\right) & \cdots & d\left({\bar{U}}_{h_{S}^{1n}},{\bar{U}}_{h_{S}^{n}}^{-}\right)\\ d\left({\bar{U}}_{h_{S}^{21}},{\bar{U}}_{h_{S}^{1}}^{-}\right) & d\left({\bar{U}}_{h_{S}^{22}},{\bar{U}}_{h_{S}^{2}}^{-}\right) & \cdots & d\left({\bar{U}}_{h_{S}^{2n}},{\bar{U}}_{h_{S}^{n}}^{-}\right)\\ \vdots & \vdots & \ddots & \vdots \\ d\left({\bar{U}}_{h_{S}^{m1}},{\bar{U}}_{h_{S}^{1}}^{-}\right) & d\left({\bar{U}}_{h_{S}^{m2}},{\bar{U}}_{h_{S}^{m}}^{-}\right) & \cdots & d\left({\bar{U}}_{h_{S}^{mn}},{\bar{U}}_{h_{S}^{n}}^{-}\right) \end{array}\right]$$

where the HFLPU-PIS $A^{+}=\{{\bar{U}}_{h_{S}^{j}}^{+}|j=1,2,\ldots n\}$ and the HFLPU-NIS $A^{-}=\{{\bar{U}}_{h_{S}^{j}}^{-}|j=1,2,\ldots n\}$ are obtained as:

(17) $${\bar{U}}_{h_{S}^{j}}^{+}=\left\{ \begin{array}{ll} \underset{i=1,2,\ldots m}{max}{\bar{U}}_{h_{S}^{ij}} & for\;benefit\;attribute\;C_{j}\left(j=1,2,\ldots ,n\right)\\ \underset{i=1,2,\ldots m}{min}{\bar{U}}_{h_{S}^{ij}} & for\;cost\;attribute\;C_{j}\left(j=1,2,\ldots ,n\right) \end{array}\right.$$

(18) $${\bar{U}}_{h_{S}^{j}}^{-}=\left\{ \begin{array}{ll} \underset{i=1,2,\ldots m}{min}{\bar{U}}_{h_{S}^{ij}} & for\;benefit\;attribute\;C_{j}\left(j=1,2,\ldots ,n\right)\\ \underset{i=1,2,\ldots m}{max}{\bar{U}}_{h_{S}^{ij}} & for\;cost\;attribute\;C_{j}\left(j=1,2,\ldots ,n\right) \end{array}\right.$$

Step 6. Calculate the relative closeness of the alternatives $A_{i}\left(i=1,2,\ldots ,m\right)$:

(19) $$RC\left(A_{i}\right)=\frac{D_{i}^{-}}{D_{i}^{+}+D_{i}^{-}}$$

where $D_{i}^{-}=\sum_{j=1}^{n}\omega_{j}d\left({\bar{U}}_{h_{S}^{ij}},{\bar{U}}_{h_{S}^{j}}^{-}\right)$, $D_{i}^{+}=\sum_{j=1}^{n}\omega_{j}d\left({\bar{U}}_{h_{S}^{ij}},{\bar{U}}_{h_{S}^{j}}^{+}\right)$ and the weights $\omega_{j}(j=1,\ldots ,n)$ are obtained by Equation (11).

Step 7. Rank the alternatives according to the relative closeness values $RC\left(A_{i}\right)\left(i=1,2,\ldots ,m\right)$ and ends the procedure. The greater the relative closeness value $RC\left(A_{i}\right)$ is, the better the alternative $A_{i}$ should be.

**Figure 3:**
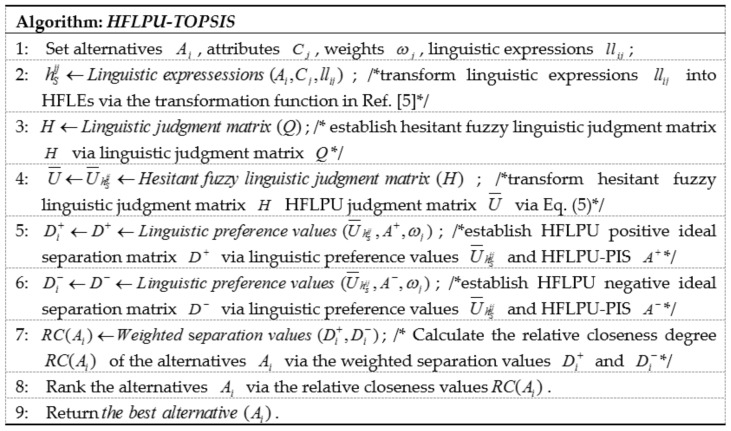
The pseudocode of the HFLPU-TOPSIS method.

The pseudocode of the HFLPU-TOPSIS method is shown in Figure 3. First, the parameters are set, including the alternatives, the attributes, the weights and the linguistic expressions, in line 1. Next, the linguistic expressions are transformed into the HFLEs in line 2. The linguistic judgment matrix and the hesitant fuzzy linguistic judgment matrix are established in lines 3, 4, respectively. Then, the HFLPU positive ideal separation matrix and the HFLPU negative ideal separation matrix are established in lines 5, 6, respectively. We calculate the relative closeness degree of the alternatives based on the weighted separation values in line 7 and then rank the alternatives in line 8. Finally, the best alternative is returned in line 9.

## 5. Case Study: Selection of Fire Rescue Plans

The DMs’ risk preference attitudes have a great effect on the response of emergency events, which are usually ignored in most studies [1,2,4]. In this section, we employ the proposed HFLPU-TOPSIS method to solve a case concerning the selection of fire rescue plans, in which different parameters are used to reflect the DMs’ risk preference attitudes. Then, some comparisons are conducted to verify the effectiveness of the proposed HFLPU-TOPSIS method.

### 5.1. Case Description

When the fire occurs, due to the uncertainty of information and the urgency of time, the DMs usually cannot make comprehensive judgments about the fire. To reduce the damage caused by the fire, many scholars developed different emergency decision methods to select the rescue plans for the fire [29,30]. To select the best fire rescue plans, three crucial factors need to consider:

- Safety coefficient. The implementations of rescue plans are related to the safety of firemen. Thus, it is necessary to choose a rescue plan with a high safety coefficient.
- Coordination degree of relevant departments. The fire poses a major threat to society and environment, which needs to be addressed by some relevant departments, such as fire department, government department and environmental protection department. Therefore, the relevant departments with a high consensus can decrease the loss caused by the fire.
- Rescue success rate. In the implementations of rescue plans, it is necessary to consider the success rate of each rescue plan. Under the same circumstance, the higher the rescue success rate is, the better the rescue plan should be.

In the following, we conduct a case study concerning the selection of emergency plans for the fire. Suppose that four rescue plans $\left\{A_{1},A_{2},A_{3},A_{4}\right\}$ are put forward to response to the fire. Three attributes $\left\{C_{1},C_{2},C_{3}\right\}$ are considered, including $C_{1}$: Safety coefficient, $C_{2}$: Coordination degree of relevant departments, $C_{3}$: Rescue success rate. Due to the uncertainty and complexity of emergency decision information, it is straightforward for the DMs to express their preferences with the linguistic expressions $ll_{ij}\left(i=1,2,3,4;j=1,2,3\right)$. The linguistic expressions $ll_{ij}(i=1,2,3,4;j=1,2,3)$ denote the possible preference degrees of the alternatives $A_{i}\left(i=1,2,3,4\right)$ over the attributes $C_{j}\left(j=1,2,3\right)$. We can see that all three attributes are benefit type attributes. The linguistic term set $S=\{s_{0}=none,s_{1}=very\;low,s_{2}=low,s_{3}=medium,s_{4}=high,s_{5}=very\;high,s_{6}=perfect\}$ can be used for these attributes. Following a heated discussion, a group of experts from relevant departments come to a consensus on the final linguistic evaluation information shown in Table 1.

### 5.2. Application of the HFLPU-TOPSIS Method

In the following, we use the proposed HFLPU-TOPSIS method to solve the above case. Different parameters are used to reflect the DMs’ risk preference attitudes. According to Equation (11), we can obtain the weights of attributes as $\omega_{1}=0.30$, $\omega_{2}=0.39$, $\omega_{3}=0.31$. The calculation procedure of the HFLPU-TOPSIS method can be listed as follows:

Step 1 and Step 2 are given above, so we go to Step 3 directly.

Step 3. According to the transformation function $E_{G_{H}}$, the linguistic judgment matrix Q can be transformed into the corresponding hesitant fuzzy linguistic judgment matrix H:

$$H=\left[\begin{array}{ccc} \left\{s_{4},s_{5}\right\} & \left\{s_{0},s_{1},s_{2}\right\} & \left\{s_{4},s_{5},s_{6}\right\}\\ \left\{s_{0},s_{1},s_{2},s_{3}\right\} & \left\{s_{4}\right\} & \left\{s_{5},s_{6}\right\}\\ \left\{s_{2},s_{3}\right\} & \left\{s_{4},s_{5}\right\} & \left\{s_{2},s_{3}\right\}\\ \left\{s_{2},s_{3}\right\} & \left\{s_{5},s_{6}\right\} & \left\{s_{0},s_{1}\right\} \end{array}\right]$$

When α=β=0.88, λ=2.25, t=3, the decision results are consistent with the empirical data [16]. Therefore, we use these parameters to address this case. According to the proposed linguistic scale function, the hesitant fuzzy linguistic judgment matrix H can be transformed into the corresponding HFLPU judgment matrix $\bar{U}$:

$$\bar{U}=\left[\begin{array}{ccc} \left\{4.00,4.84\right\} & \left\{-2.92,-1.14,0.75\right\} & \left\{4.00,4.84,5.63\right\}\\ \left\{-2.92,-1.14,0.75,3.00\right\} & \left\{4.00\right\} & \left\{4.84,5.63\right\}\\ \left\{0.75,3.00\right\} & \left\{4.00,4.84\right\} & \left\{0.75,3.00\right\}\\ \left\{0.75,3.00\right\} & \left\{4.84,5.63\right\} & \left\{-2.92,-1.14\right\} \end{array}\right]$$

Step 4. According to the score function and the variance function in Definition 6, we can obtain ${\bar{U}}_{H_{S}^{1}}^{+}={\bar{U}}_{H_{S}^{11}}=\{4.00,4.84\}$, ${\bar{U}}_{H_{S}^{2}}^{+}={\bar{U}}_{H_{S}^{42}}=\{4.84,5.63\}$, ${\bar{U}}_{H_{S}^{3}}^{+}={\bar{U}}_{H_{S}^{23}}=\{4.84,5.63\}$, ${\bar{U}}_{H_{S}^{1}}^{-}={\bar{U}}_{H_{S}^{21}}=$ {−2.92,−1.14,0.75,3.00}, ${\bar{U}}_{H_{S}^{2}}^{-}={\bar{U}}_{H_{S}^{12}}=\{-2.92,-1.14,0.75\}$, ${\bar{U}}_{H_{S}^{3}}^{-}={\bar{U}}_{H_{S}^{43}}=\{-2.92,-1.14\}$, respectively. So the HFLPU-PIS $A^{+}$ and the HFLPU-NIS $A^{-}$ can be obtained as:

$$A^{+}={\left(\left\{4.00,4.84\right\},\left\{4.84,5.63\right\},\left\{4.84,5.63\right\}\right)}^{T};$$

$$A^{-}={\left(\left\{-2.92,-1.14,0.75,3.00\right\},\left\{-2.92,-1.14,0.75\right\},\left\{-2.92,-1.14\right\}\right)}^{T}$$

Step 5. The positive ideal separation matrix $D^{+}$ and the negative ideal separation matrix $D^{-}$ can be established by Equation (9), shown below:

$$D^{+}=\left[\begin{array}{ccc} 0.00 & 0.75 & 0.06\\ 0.57 & 0.26 & 0.00\\ 0.30 & 0.09 & 0.40\\ 0.30 & 0.00 & 0.68 \end{array}\right],\;D^{-}=\left[\begin{array}{ccc} 0.57 & 0.00 & 0.63\\ 0.00 & 0.50 & 0.68\\ 0.28 & 0.66 & 0.29\\ 0.29 & 0.75 & 0.00 \end{array}\right]$$

Step 6. By Equation (19), we can obtain the weighted Euclidean distances as $D_{1}^{+}=0.31$, $D_{2}^{+}=0.27$, $D_{3}^{+}=0.25$, $D_{4}^{+}=0.30$, $D_{1}^{-}=0.44$, $D_{2}^{-}=0.40$, $D_{3}^{-}=0.50$, $D_{4}^{-}=0.41$ and then obtain the relative closeness degrees of the rescue plans as $RC\left(A_{1}\right)=0.54$, $RC\left(A_{2}\right)=0.60$, $RC\left(A_{3}\right)=0.63$, $RC\left(A_{4}\right)=0.56$.

Step 7. According to the values of the relative closeness $RC\left(A_{i}\right)\left(i=1,2,3,4\right)$, the ranking of fire rescue plans is $A_{3}≻A_{2}≻A_{4}≻A_{1}$. Thus, the best fire rescue plan is $A_{3}$.

### 5.3. Sensitive Analysis

In the above solving process, we only calculate the ranking result of the fire rescue plans with the risk preference parameters α=β=0.88, λ=2.25, t=3. To reflect the DMs’ risk preference attitudes, different parameters are used to solve this problem. Suppose that t=3. The decision results with different risk preference parameters are shown in Table 2.

**Figure 4:**
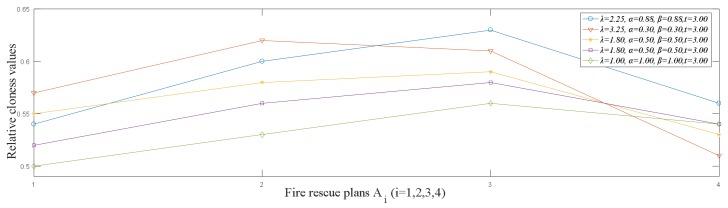
The ranking results obtained by different risk preference parameters.

From Table 2, we can find that the ranking results of rescue plans are related to the risk preference parameters. When different parameters are used, the obtained ranking results of fire rescue plans are slightly different. To have a clear view of the differences among the ranking results of fire rescue plans in Table 2, we use Figure 4 to depict the changes of these ranking results. Given that the obtained ranking results are different as the parameter changes, the DMs’ risk preference attitudes have an important impact on the selection of fire rescue plans. With the proposed HFLPU-TOPSIS method, the DMs can use different parameters to select the fire rescue plan that conform to their cognition.

### 5.4. Comparative Analysis

To illustrate the validity of the HFLPU-TOPSIS method, in the following, we compare it with two relevant MADM methods, including the HFL-TOPSIS method [31] and the HFL-VIKOR method [8].

(1) Comparison with the HFL-TOPSIS method

In this subsection, the HFL-TOPSIS method [31] is used to solve the case. Firstly, we need to add some linguistic terms into the short HFLTSs in H according the method proposed in Ref. [6]. For example, the linguistic term $s_{4.5}$ can be added into the short HFLE $\left\{s_{4},s_{5}\right\}$. Subsequently, according to the method proposed in Ref. [31], we obtain the HFL-PIS $A^{+}=\{[4.0,4.5,4.5,5.0],[5.0,5.5,5.5,6.0],[5.0,5.5,5.5,6.0]\}$ and the HFL-NIS $A^{-}=\{[0.0,1.0,2.0,3.0],[0.0,1.0,1.0,2.0],[1.0,1.5,1.5,2.0]\}$, respectively. According to the distance measure $d(H_{S}^{1},H_{S}^{2})=|q^{\prime }-q|+|p^{\prime }-p|$ defined in Ref. [31], we obtain the positive ideal separation matrix $D^{+}$ and the negative separation matrix $D^{-}$ as:

$$D^{+}=\left[\begin{array}{ccc} 0.00 & 18.00 & 2.00\\ 12.00 & 10.00 & 0.00\\ 8.00 & 2.00 & 12.00\\ 8.00 & 0.00 & 16.00 \end{array}\right],\;D^{-}=\left[\begin{array}{ccc} 12.00 & 0.00 & 14.00\\ 0.00 & 8.00 & 16.00\\ 4.00 & 16.00 & 4.00\\ 4.00 & 18.00 & 0.00 \end{array}\right]$$

**Figure 5:**
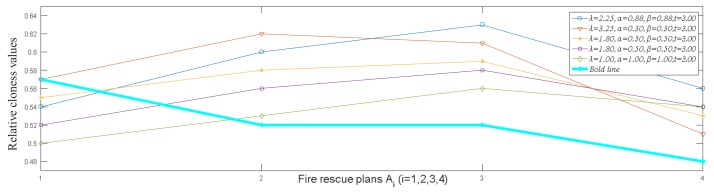
The diversity of the decision results obtained by different methods. Note. The bold line denotes the decision result obtained by the HFL-TOPSIS method [31].

Since $RC\left(A_{i}\right)=D_{i}^{-}/\left(D_{i}^{+}+D_{i}^{-}\right)$, we obtain the relative closeness degrees of the alternatives as $RC\left(A_{1}\right)=0.57$, $RC\left(A_{2}\right)=0.52$, $RC\left(A_{3}\right)=0.52$, $RC\left(A_{4}\right)=0.48$. Thus, the ranking result of fire rescue plans is $A_{1}≻A_{2}=A_{3}≻A_{4}$. That is to say, the best alternative is $A_{1}$, which is different from the decision results obtained by the proposed HFLPU-TOPSIS method. The diversity of the decision results obtained by the proposed HFLPU-TOPSIS method and the HFL-TOPSIS method [31] can be illustrated in Figure 5.

Comparative analysis: From Figure 5, we can see that the ranking results obtained by the HFL-TOPSIS method [31] are different from those obtained by the proposed HFLPU-TOPSIS method. Moreover, according to Figure 5, the relative closeness degrees of alternatives $A_{2}$ and $A_{3}$ are equal. The comparative analysis for these different results is outlined as: (1) in practical decision-making process, the information regarding to different attributes are usually different. However, the method in Reference [31] assumes that the attributes have equal importance, which does not consider the differences among attributes. In this paper, we propose a weight-determining method that assigns the weights to attributes according to the deviations between attributes; (2) the method in Reference [31] only uses the subscripts of the maximum and minimum linguistic terms in the HFLTS to calculate the distances between different HFLTSs, which leads to the loss of information. Most importantly, only one type of ranking result can be obtained by the HFL-TOPSIS method, which cannot reflect the DMs’ risk preference attitudes comprehensively. By contrast, using the proposed linguistic scale function, we can transform the linguistic terms into the corresponding linguistic preference values to represent the DMs’ risk preference attitudes. Besides, it can accurately represent the semantics of linguistic terms and obtain the decision results that are consistent with the DMs’ cognition.

(2) Comparison with the HFL-VIKOR method

We can also use the HFL-VIKOR method [8] to solve the above case. According to the dispersion of attribute information calculated by the subscripts of linguistic terms, we obtain the weights of attributes as $\omega_{1}=0.28$, $\omega_{2}=0.40$, $\omega_{3}=0.32$. By the HFL-VIKOR method [8], the fuzzy linguistic group utility degrees $HFLGU_{i}\left(i=1,2,3,4\right)$, the hesitant fuzzy linguistic individual regret degrees $HFLIR_{i}\left(i=1,2,3,4\right)$ and the hesitant fuzzy linguistic compromise degrees $HFLC_{i}\left(i=1,2,3,4\right)$ can be calculated by:

(20) $$HFLIR_{i}=\max\left(\omega_{j}\frac{d_{ed}(H_{j}^{+},H_{j}^{i})}{d_{ed}(H_{j}^{+},H_{j}^{-})}\right)$$

(21) $$HFLIR_{i}=\max\left(\omega_{j}\frac{d_{ed}(H_{j}^{+},H_{j}^{i})}{d_{ed}(H_{j}^{+},H_{j}^{-})}\right)$$

(22) $$HFLC_{i}=\theta \frac{HFLGU_{i}-HFLGU^{+}}{HFLGU^{-}-HFLGU^{+}}+(1-\theta )\frac{HFLIR_{i}-HFLIR^{+}}{HFLLIR^{-}-HFLIR^{+}}$$

Without loss of generality, we set the maximum overall utility parameter θ=0.5. Then, according to Equations (20)–(22), we can obtain the computational results as shown in Table 3.

In Table 3, $d_{*}\left(h_{s}^{ij},h_{s}^{j+}\right)$ is the Euclidean distance between each evaluated value $h_{s}^{ij}$ and the HFL-PIS $h_{s}^{j+}$. From Table 3, the ranking results of $HFLC_{i}\left(i=1,2,3,4\right)$, $HFLIR_{i}\left(i=1,2,3,4\right)$, $HFLGU_{i}\left(i=1,2,3,4\right)$ can be obtained as $HFLC_{3}<HFLC_{1}<HFLC_{2}<HFLC_{4}$, $HFLIR_{3}<HFLIR_{2}<HFLIR_{4}<HFLIR_{1}$, $HFLGU_{1}<HFLGU_{3}<HFLGU_{2}<HFLGU_{4}$. Unfortunately, according to the comparison operations [8], we cannot find the best possible compromise solution.

Comparative analysis: Although the HFL-VIKOR method [8] is very useful in tackling MADM problems in circumstances that are attributed to conflict with each other, it has the following limitations: (1) In some cases, the best solution or the ranking results of alternatives cannot be obtained by this method. Especially, when the number of alternatives is large, it is difficult to find the best alternative or rank these alternatives. By contrast, the proposed method can address more comprehensive and complex problems; (2) Similar to the HFL-TOPSIS method [31], the HFL-VIKOR method also ignores the DMs’ risk preference attitudes, which leads to the obtained decision results that are not consistent with the DMs’ cognition. Nevertheless, the proposed method can employ the hesitant fuzzy linguistic preference utility values to reflect DMs’ risk preference attitudes, which is feasible and effective to deal with emergency events.

In summary, according to the comparative analysis above, the benefits and disadvantages of the proposed methods can be listed as follows:

(1) The amount of information is one of the key factors in responding to emergency events. In general, the greater the amount of information provided by attributes is, the more accurate the decision results would be. To obtain valuable information in emergency situations and overcome the instability of subjective weights, according to the diversity of attribute information, we developed a method to obtain objective weights within the HFLPU context.
(2) To accurately reflect the DMs’ subjective feelings, we proposed the linguistic scale function to transform the semantics corresponding to the linguistic terms into the linguistic preference values. According to the proposed linguistic scale function, we proposed the HFLPUS to flexibly express the semantics and obtain the decision results that are consistent with the DMs’ cognition. Due to DMs’ knowledge and experience are various, different DMs tend to have different risk preference attitudes. Thus, when using the proposed linguistic scale function, it is necessary to select the parameters that conform to DMs’ risk preference attitudes.
(3) For calculations and comparisons, we defined the Euclidean distance measure and comparison laws for HFLPUSs. Compared with the existing methods, the defined Euclidean distance measure and comparison laws take into account of the DMs’ risk preference attitudes, which can obtain the decision results that are consistent with the DMs’ cognition.
(4) To respond to emergency events, we proposed the HLPU-TOPSIS method based on the HFLTSs and the TOPSIS method and applied it to select the best fire rescue plan. Compared with the HFL-TOPSIS [31] and the HFL-VIKOR method [8], the proposed HFLPU-TOPSIS method can not only deal with the MADM problems in emergency situations but also considers the DMs’ risk preference attitudes by using different parameters. However, it is noted that DMs should come to consensus about the decision information of alternatives with respect to attributes. Thus, the proposed method is not feasible when the DMs conflict with each other.

## 6. Conclusions

In this paper, we proposed the linguistic scale function to transform the linguistic term into the linguistic preference values. Based on that, we developed the HFLPUS to obtain decision results that are consistent with the DMs’ cognition. Additionally, by using risk preference parameters, the HFLTS can be transformed into the corresponding HFLPUS. For calculations and comparisons, we defined the distance measure and comparison laws for HFLPUSs under linguistic preference value environment. For application, we developed the HFLPU-TOPSIS method based on the classical TOPSIS method and the HFLTSs. To overcome the instability of subjective weights, a method to determine objective weights was introduced according to the diversity of attribute information. Finally, to illustrate the validity of HFLPU-TOPSIS method, some comparisons with other two representative MADM methods were conducted. The comparison results showed that the proposed HFLPU-TOPSIS method can not only deals with the MADM problems but also allows the DMs to make decision according to their risk preference attitudes.

In the future, we will continue our research from the following research directions: (1) Based on the proposed linguistic scale function, we shall study some new linguistic computing models and aggregation operators that are consistent with the DMs’ cognition. In addition, based on the subscript-symmetric linguistic term set, some novel linguistic scale functions may be further developed; (2) The proposed HFLPUS can not only elicit linguistic evaluation information but also considers the DMs’ risk preference attitudes, based on which, some other MADM methods and aggregation operators may be developed in future studies; (3) The proposed HFLPU-TOPSIS method can be applied to solve the MADM problems in other fields, such as artificial intelligence, supply chain management, medical diagnosis. In addition, it may be extended to address the group MADM problems.

## Figures and Tables

**Table 1:** The linguistic expressions provided by the DMs.

	$C_{1}$	$C_{2}$	$C_{3}$
$A_{1}$	between high and very high	at most low	at least high
$A_{2}$	at most medium	high	at least very high
$A_{3}$	between low and medium	between high and very high	between low and medium
$A_{4}$	between low and medium	at least very high	between very low and low

**Table 2:** The ranking results obtained by different risk preference parameters.

	$RC\left(A_{1}\right)$	$RC\left(A_{2}\right)$	$RC\left(A_{3}\right)$	$RC\left(A_{4}\right)$	**Ranking Results**
α=β=0.88,λ=2.25	0.54	0.60	0.63	0.56	$A_{3}≻A_{2}≻A_{4}≻A_{1}$
α=0.30,β=0.30,λ=3.25	0.57	0.62	0.61	0.51	$A_{2}≻A_{3}≻A_{1}≻A_{4}$
α=0.50,β=0.50,λ=1.80	0.55	0.58	0.59	0.53	$A_{3}≻A_{2}≻A_{1}≻A_{4}$
α=β=0.70,λ=1.50	0.52	0.56	0.58	0.54	$A_{3}≻A_{2}≻A_{4}≻A_{1}$
α=β=λ=1.00	0.50	0.53	0.56	0.54	$A_{3}≻A_{4}≻A_{2}≻A_{1}$

**Table 3:** The decision-making results obtained by the HFL-VIKOR method.

	$d_{*}\left(h_{s}^{i1},h_{s}^{1+}\right)$	$d_{*}\left(h_{s}^{i2},h_{s}^{2+}\right)$	$d_{*}\left(h_{s}^{i3},h_{s}^{3+}\right)$	$HFLGU_{i}$	$HFLIR_{i}$	$HFLC_{i}$
$A_{1}$	0.00	0.75	0.10	0.45	0.40	0.50
$A_{2}$	0.52	0.42	0.00	0.51	0.28	0.63
$A_{3}$	0.33	0.12	0.50	0.48	0.24	0.26
$A_{4}$	0.33	0.00	0.67	0.50	0.32	0.65
